# Effect of Three Different Denture Cleansers on the Impact Strength of Heat-Cure Polymethylmethacrylate and Polyamide Denture Base Resin: An In Vitro Study

**DOI:** 10.7759/cureus.92894

**Published:** 2025-09-22

**Authors:** Nissy Chacko, Kala Sukumaran

**Affiliations:** 1 Dentistry, Government Dental College Thiruvananthapuram, Thiruvananthapuram, IND

**Keywords:** clinsodent, denture base resin, denture cleanser, impact strength, polident, polyamide, polymethylmethacrylate, valclean

## Abstract

Background

This in vitro study aimed to evaluate and compare the effects of three commercially available denture cleansers on the impact strength of two common denture base resins, namely, heat-polymerized polymethylmethacrylate (PMMA) and polyamide. The cleansers used in the study included Valclean (acidic), Polident (neutral), and Clinsodent (alkaline).

Methodology

A total of 112 specimens from two groups (Group A and B) with 14 specimens in eight subgroups were made as per ISO 1567:1999/Amd. 1:2003(E). Group A consisted of 56 PMMA (Acryton H) specimens, while Group B consisted of 56 polyamide (Valplast) specimens. Each primary group was categorized into the following four subgroups: distilled water - control (A1 and B1), Valclean (A2 and B2), Polident (A3 and B3), and Clinsodent (A4 and B4). All specimens underwent a six-month immersion schedule, alternating between distilled water and the cleanser solutions. A Charpy-type digital impact testing machine was used to measure the impact strength, and statistical analysis was conducted. Quantitative parameters were compared among the categories using a one-way analysis of variance (F test).

Results

Both heat-polymerized PMMA (Acryton H) and polyamide (Valplast) showed a statistically significant reduction in impact strength following immersion in the denture cleansers used in this study (p < 0.01). The reduction in strength was progressively evident across cleanser groups, indicating that prolonged cleanser exposure can significantly alter the mechanical integrity of denture base resins.

Conclusions

The impact strength of both PMMA and polyamide resins was affected by immersion in denture cleaners. Cleansers with extreme pH levels, both acidic and alkaline, led to greater material degradation, while the neutral cleanser (Polident) caused minimal changes. These findings underscore the significance of selecting denture cleansers carefully, especially for prolonged use.

## Introduction

Polymethylmethacrylate (PMMA) and polyamide are widely used denture base materials in prosthodontics due to their favorable mechanical properties, cost-effectiveness, and patient comfort. PMMA has long been regarded as the material of choice for denture bases because of its acceptable strength, aesthetic properties, and ease of fabrication. In contrast, thermoplastic polyamide resins have gained popularity in recent years for their flexibility, improved impact resistance, and adaptability, especially in patients with compromised ridges or allergies to acrylic monomers. Denture hygiene is essential for oral health, and while mechanical cleaning remains the primary recommendation, chemical denture cleansers are often used as an adjunct or alternative, particularly among elderly or physically impaired individuals. Numerous studies have evaluated the effects of denture cleansers on surface roughness, color stability, and microbial reduction of denture base resins; however, few studies have focused on their influence on mechanical properties such as impact strength [1-3]. A reduction in impact strength increases the risk of denture fracture.

Elderly or medically compromised patients rely on chemical cleansers for maintaining denture hygiene. Although current literature indicates that prolonged exposure to denture cleansers may alter the surface characteristics of denture base resins [4,5], the extent to which different pH-based cleansers affect their impact strength remains inadequately explored. Alkaline peroxide cleansers, in particular, have been reported to degrade the polymer matrix over time due to oxidative breakdown. Acidic or neutral cleansers may interact differently with resin components, depending on their chemical formulations. Moreover, the structural differences between PMMA and polyamide resins, including their polymer network, water sorption rates, and residual monomer content, contribute to variable responses upon exposure. These inconsistencies highlight a gap in comparative data that assesses how commercial denture cleansers, especially with varied pH levels, impact the mechanical integrity of different denture base materials.

This in vitro study aimed to evaluate and compare the effects of three commercially available denture cleansers, namely, Valclean (acidic), Polident (neutral), and Clinsodent (alkaline), on the impact strength of heat-polymerized PMMA (Acryton H) and polyamide (Valplast) denture base resins. By simulating six-month cleanser exposure, this study attempts to provide insights into the compatibility of these cleaning agents with different denture materials, helping clinicians make evidence-based decisions in recommending appropriate cleansers for long-term use. The study contributes to existing knowledge by addressing a poorly explored aspect of denture maintenance: the mechanical degradation of denture base materials following chemical cleanser immersion.

## Materials and methods

Specimen preparation

**Figure 1:**
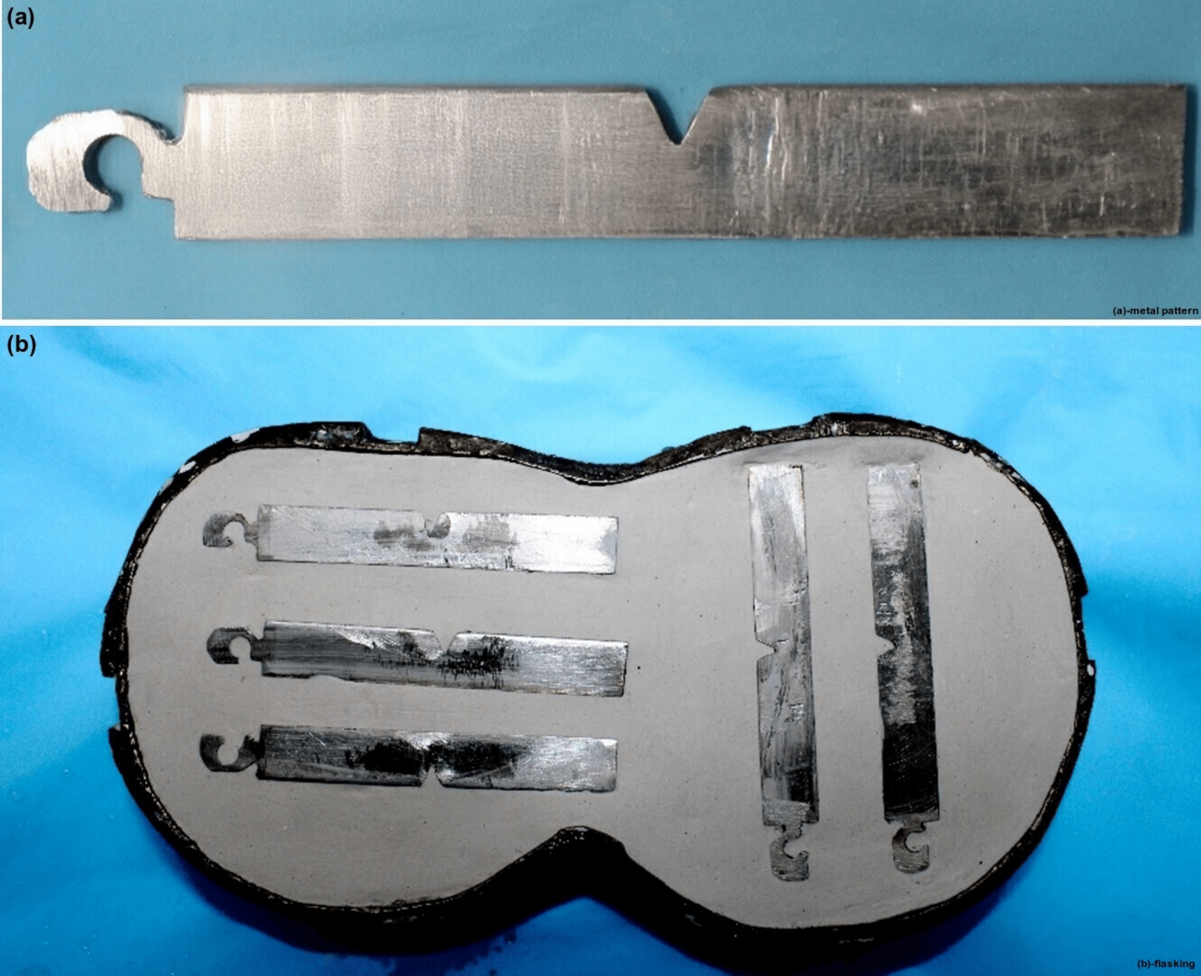
Metal pattern and investing. (a) Metal Pattern as per the ISO 1567:1999/Amd.1:2003(E). (b) Investment of the metal pattern.

A metal die of dimensions 52 × 50 × 4 mm (length, width, and thickness) with a 2 mm V-notch in the middle following ISO 1567 was created (Figure 1a). Additionally, a 4 mm hook was added at one end to facilitate easy removal from the flask. When preparing the mold, the traditional flasking method for full dentures was used. Following the manufacturer’s instructions, a fresh mix of model plaster (with a 25 g/100 g water-to-powder ratio) was added to the lower part of the denture flask, and the metal patterns were invested (Figure 1b).

**Figure 2:**
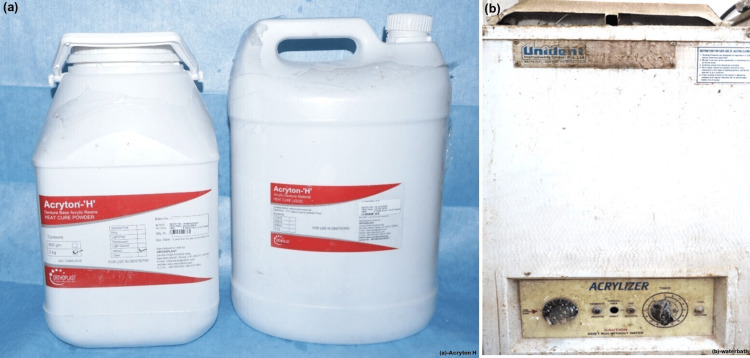
Acryton H and waterbath. (a) Acryton H: heat-polymerized polymethylmethacrylate. (b) Water bath for polymerization.

Separating media was then applied. The upper half of the flask was placed on top of the bottom half and filled with plaster. The metal dies were removed after the plaster had set. Acryton H is a traditional heat-cure denture base material with a powder-to-liquid ratio of 24 g/10 mL (Figure 2a). During the dough stage, the material was packed in accordance with the manufacturer’s instructions. After maintaining pressure for five minutes, the flask was kept for curing. The clamped flasks were cured by submerging them in the water bath (Figure 2b) and heating them to 74°C for around 90 minutes.

**Figure 3:**
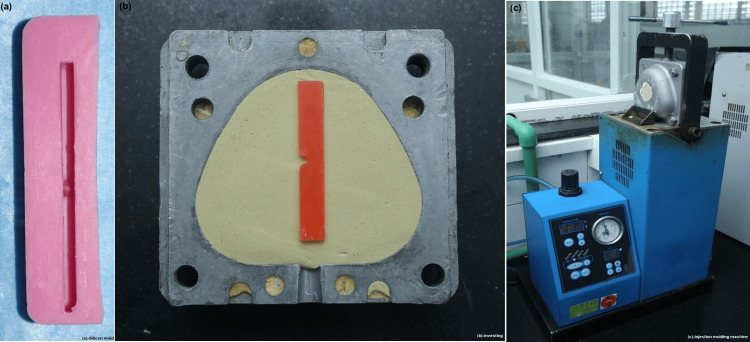
Silicon mold, investing the wax replica, and the injection molding machine. (a) Silicon mold for Valplast sample fabrication. (b) Investing wax pattern made from the silicon mold. (c) Flask placed in the injection molding machine.

According to ADA 1, the temperature was increased to boil and held for 30 minutes. Following curing, the clamped flasks were submerged in water for 15 minutes and then allowed to cool at ambient temperature for 30 minutes. The acrylic samples were taken out of the mold, the hook section was cut, and the pattern was sanded using sequential grits of sandpaper (80, 320, 400, and 1,000) and polished with pumice. A Zoom India electronic digital caliper with an accuracy of 0.02 mm/0.001 inch was used to measure each sample’s length, width, and thickness. A total of 56 Acryton H samples were prepared in a similar fashion. After that, the samples were kept for 24 hours in distilled water. For Valplast specimens, a silicone mold (Figure 3a) was fabricated using a reference die of predetermined dimensions similar to that of PMMA. Modelling wax was added to the silicone mold to obtain the desired form. The required number of wax replicas was fabricated using the mold. The wax samples were flasked in an injection molding flask using a dental stone-plaster mixture (Figure 3b). Following the application of a separating medium, counter pouring was done using dental plaster. Dewaxing was performed to eliminate the wax pattern from the mold. The flask was opened, and steam cleaning was performed to remove any residual wax. A separating medium was applied to the mold surface to facilitate easy retrieval of the final sample. The injection molding material cartridge was loaded into the injection molding machine (Figure 3c).

**Figure 4:**
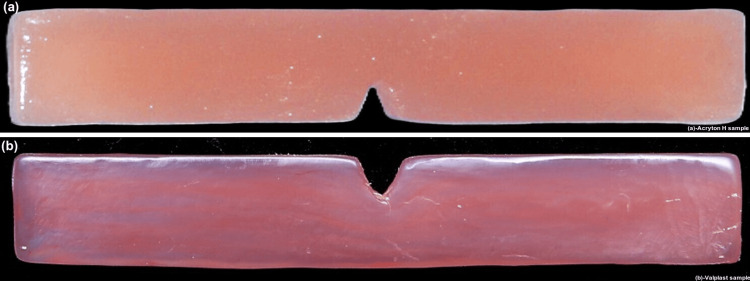
Acryton H and Valplast samples. (a) Acryton H sample. (b) Valplast sample.

The flask was then closed and securely clamped to the machine. Injection molding was done to transfer the material into the flask under controlled pressure and temperature conditions. Once the injection molding process was complete, the flask and cartridge were carefully taken out of the machine. The support sprue was cut, and the specimen was finished and polished using carbide, stone, and diamond burs, progressing from higher to lower abrasiveness. The process was followed by wet and dry buffing to attain a smooth and polished finish. A total of 56 Valplast samples were prepared adopting the same method (Figures 4a, 4b).

Immersion procedure

**Figure 5:**
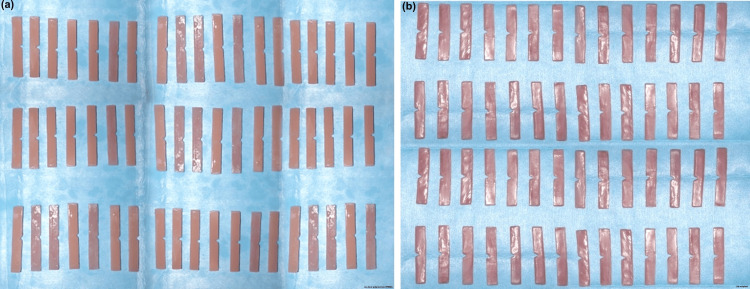
Group A (Acryton H) and Group B (Valplast) samples. (a) Group A: heat-polymerized polymethylmethacrylate (Acryton H). (b) Group B: Valplast.

The samples were categorized into two major groups, namely, Group A (Acryton H) and Group B (Valplast), with 56 samples in each group (Figures 5a, 5b).

The polymerization technique and composition of the materials are outlined in Table 1.

**Table 1:** Denture base materials.

Groups	Trade name	Polymerization technique	Composition
A	Acryton H (Orthoplast - NID)	Compression molding	Powder: polymethylmethacrylate beads. Initiator: benzoyl peroxide pigment: salt of cadmium/iron or organic dye. Liquid: monomethylmethacrylate (monomer). Cross-linking agent: ethylene glycol dimethacrylate Initiator. Hydroquinone activator: N,N dimethyl P-toluedine
B	Valplast (Valplast Int. Corp.)	Injection molding	Polyamide-12 (Nylon resins). Monomers: caprolactam, ethylene glycol dimethacrylate, triethylene glycol dimethacrylate, methacrylic acid. Plasticizers additives. Coloring agent. UV stabilizer

**Figure 6:**
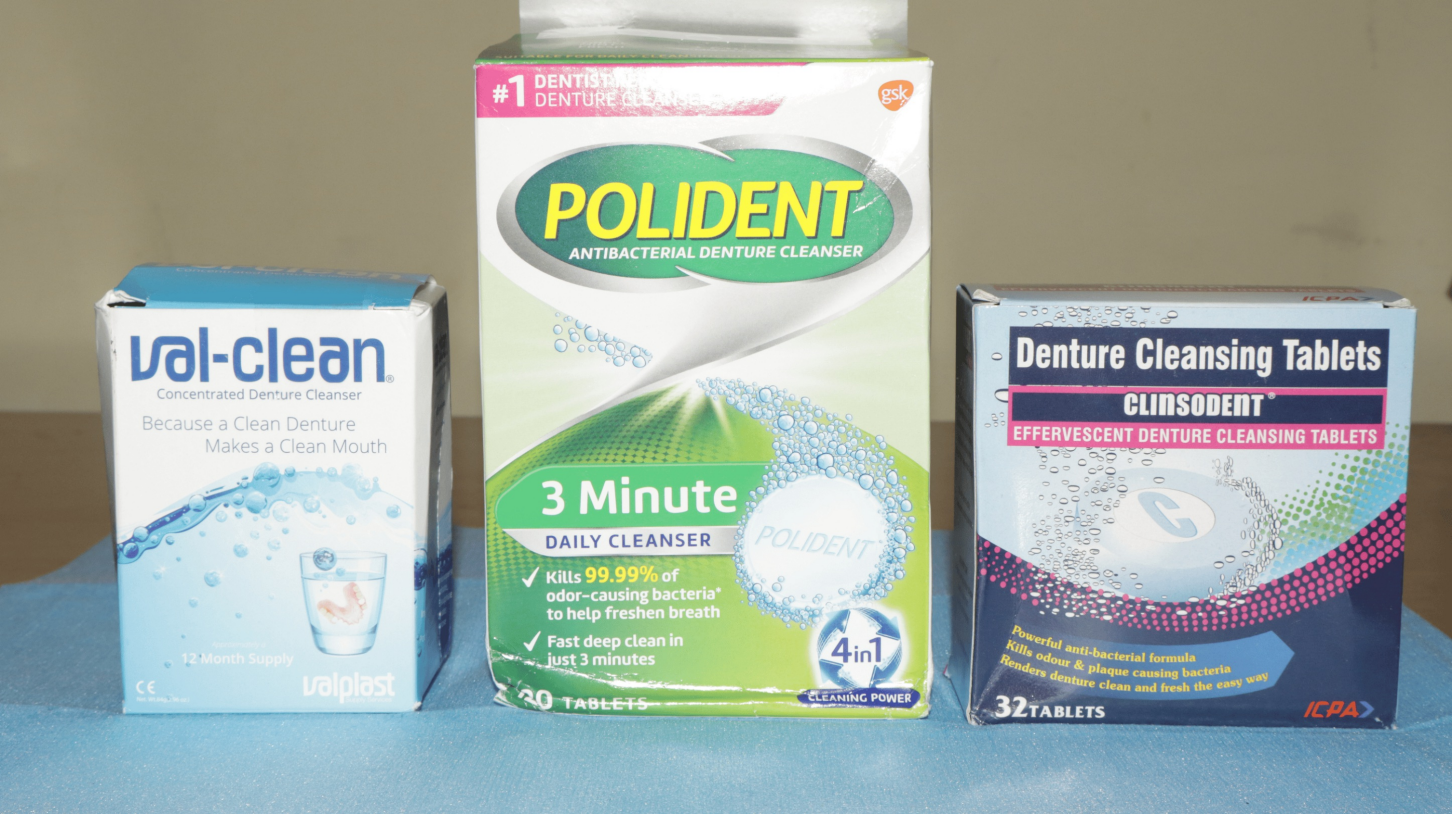
Denture cleansers.

The chosen denture cleansers were Valclean, Polident, and Clinsodent (Figure 6), and the composition and pH of the cleansers are summarized in Table 2.

**Table 2:** Composition and pH of denture cleansers.

Denture cleanser	Composition	pH
Valclean	Sodium hypochlorite, potassium peroxymonopersulfate, citric acid, potassium bisulfate, magnesium carbonate, potassium sulfate, peppermint extract, potassium peroxydisulphate, sucrose	Acidic
Polident	Sodium perborate, sodium bicarbonate, citric acid, potassium caroate (potassium monopersulfate), sodium carbonate, sodium carbonate peroxide, TAED, sodium benzoate, PEG-180, sodium lauryl Sulfate, VP/VA copolymer, aroma, cellulose gum, CI 42090, CI 73015, CI 19140	Neutral
Clinsodent	Sodium perborate, sodium bicarbonate, potassium persulfate. inactive ingredients: sodium carbonate, sodium sulfate, trisodium phosphate, sulphamic acid, tetra potassium pyro phosphate, EDTA, sodium lauryl sulfate, peppermint powder, etc.	Alkaline

**Figure 7:**
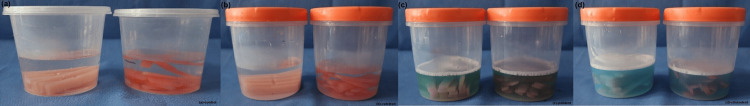
Samples immersed in solutions. (a) Control group (heat-cured polymethylmethacrylate and Valplast samples in distilled water). (b) Valclean. (c) Polident. (d) Clinsodent.

The samples in each group were randomly divided into four equal subgroups (Figure 7), namely, A1 and B1 (control - distilled water), A2 and B2 (Valclean), A3 and B3 (Polident), and A4 and B4 (Clinsodent).

**Figure 8:**
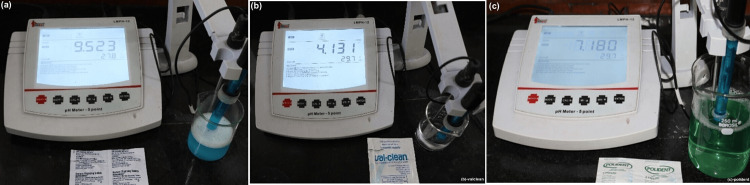
pH meter showing the pH of denture cleansers. (a) Clinsodent with alkaline pH (9.523). (b) Valclean with acidic pH (4.131). (c) Polident with neutral pH (7.180).

Denture cleanser immersion was done as per the manufacturer’s instructions. The samples were submerged in distilled water between the cycles. One Clinsodent (ICPA) tablet (480 mg) was diluted in 50 mL of warm water and the samples were immersed for 30 minutes; one teaspoonful of Valclean liquid denture cleanser was diluted with 50 mL of water and the samples were immersed for 30 minutes; and one Polident tablet was diluted in 250 mL of warm water and the samples were immersed for three minutes. Immersion was done for three months, simulating a six-month immersion. There was a 12-hour interval between two successive cycles of immersion, and specimens were immersed in distilled water during this time. Freshly prepared cleanser solution was used for each cycle. pH measurement of the cleansers was done using a pH meter (Figure 8).

Testing the impact strength

**Figure 9:**
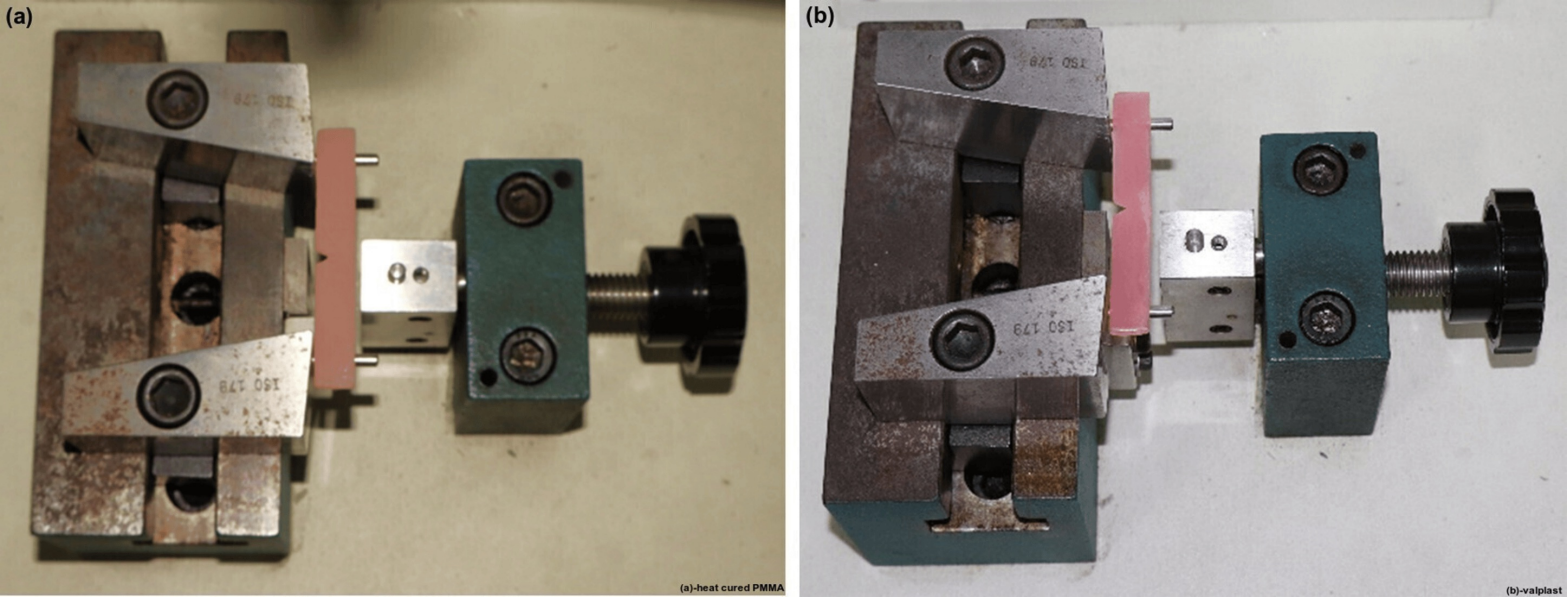
Samples placed in position for testing. (a) Heat-cured polymethylmethacrylate. (b) Valplast.

Testing was done by using a Charpy-type digital impact testing machine (Tinius Olsen Model Impact 503). In total, 14 specimens of eight subgroups were used for impact strength determination (n = 112). The test specimens were placed on the platform of the impact testing machine with their unnotched side facing away from the pendulum (Figure 9).

**Figure 10:**
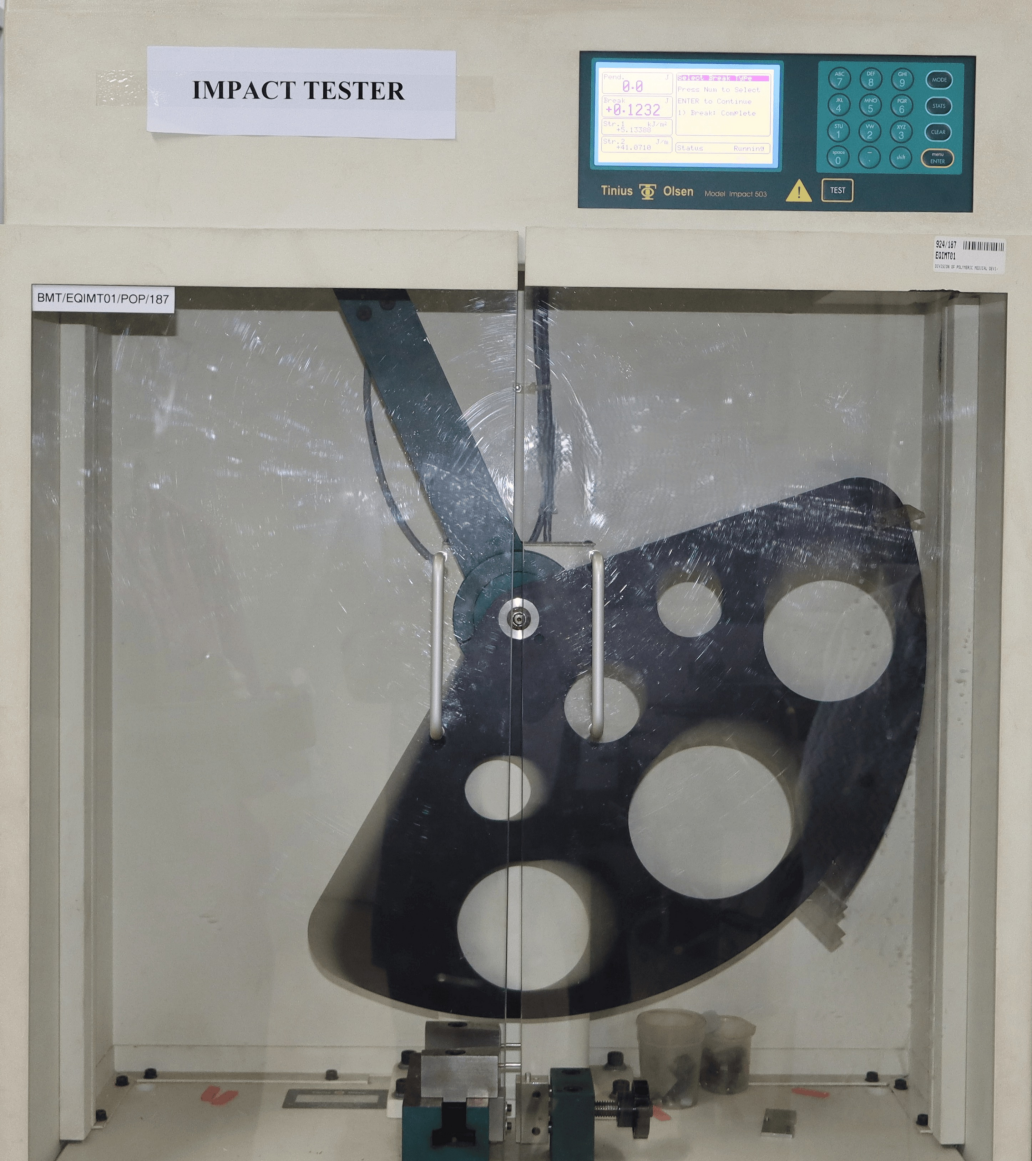
Testing the samples using the Charpy impact tester.

A pendulum of 2 J testing capacity was used, and the impact speed of the pendulum was 3.46 m/second. The pendulum was released, which swung down to fracture the center of the bar specimen supported at both ends (Figure 10).

The energy lost by the pendulum during the fracture of the specimen could be determined by a comparison of the length of its swing after the impact with that of its free swing when no impact occurs. The energy absorbed by the test specimen at the time of fracture was given by a calibrated scale. Impact strength was calculated using the following formula and expressed in kJ/m^2^: 𝐸/𝐴, where E = energy absorbed by specimen at breaking and A = area of cross-section (30 mm^2^).

Statistical analysis

Quantitative data were summarized using mean ± standard deviation (SD), median with interquartile range (IQR), and minimum-maximum values. A one-way analysis of variance (ANOVA) was performed to compare the mean impact strength among the four subgroups within each material category (PMMA and polyamide). A p-value of less than 0.05 was considered statistically significant. Statistical analysis was performed using SPSS Statistics, Version 20.0 (IBM Corp., Armonk, NY, USA). Results were also presented graphically using box plots for visual comparison.

## Results

Following immersion in different denture cleansers, both PMMA (Acryton H) and polyamide (Valplast) specimens demonstrated statistically significant reductions in impact strength (p < 0.01). The progressive decline in impact strength from control to acidic, neutral, and alkaline cleanser groups was evident. These variations are summarized in Table 3 and Table 4.

**Table 3:** Descriptive statistics of impact strength of samples in the four different subgroups. Categorical and quantitative variables are expressed as frequency (percentage) and mean ± SD, respectively. Descriptive statistics such as mean ± SD, median with interquartile range, minimum, and maximum are used to describe impact strength.

Heat-polymerized polymethylmethacrylate (Acryton H)				
Group	Mean ± SD	Median (IQR)	Minimum	Maximum
A1	1.73 ± 0.4	1.73 (1.53–1.94)	1.02	2.51
A2	1.23 ± 0.34	1.1 (0.97–1.54)	0.87	1.95
A3	0.95 ± 0.22	0.93 (0.8–1.1)	0.56	1.34
A4	0.86 ± 0.14	0.88 (0.77–0.98)	0.56	1.03
Polyamide (Valplast)				
B1	367.2 ± 5.4	366.37 (364.33–368.36)	356.7	378.5
B2	346.8 ± 4.7	346.03 (342.34–350)	342.1	355.3
B3	331.2 ± 5.4	330.72 (327.68–335.73)	321.6	342.1
B4	305.1 ± 6.8	305.05 (298.75–309.96)	297.6	319.6

**Table 4:** Comparison of impact strength of samples in the four different subgroups. Categorical variables are represented as frequency (percentage). One-way analysis of variance (F test) was used to compare quantitative parameters between categories. For all statistical interpretations, p < 0.05 was considered the threshold for statistical significance.

Heat-polymerized polymethylmethacrylate (Acryton H)					
Group	Mean	SD	N	F	P-value
A1	1.73	0.40	14	24.42	<0.01
A2	1.23	0.34	14		
A3	0.95	0.22	14		
A4	0.86	0.14	14		
Polyamide (Valplast)					
B1	367.2	5.4	14	305.42	<0.01
B2	346.8	4.7	14		
B3	331.2	5.4	14		
B4	305.1	6.8	14		

**Figure 11:**
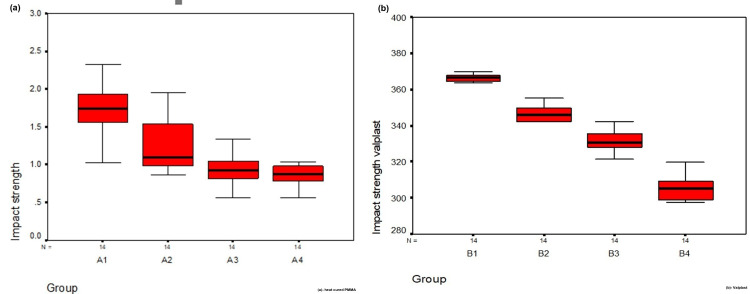
Box plot of the impact strength of heat-polymerized polymethylmethacrylate (Acryton H) and Polyamide (Valplast) in the four different subgroups. Descriptive statistics such as mean ± SD, median with interquartile range, minimum, and maximum are used to describe impact strength. A box plot was used for the graphical presentation of descriptive summary statistics such as minimum, first quartile, median, third quartile, and maximum. (a) Box plot for heat-cured polymethylmethacrylate. (b) Box plot for Valplast. A1, B1: control groups; A2, B2: Valclean; A3, B3: Polident; A4, B4: Clinsodent.

**Figure 12:**
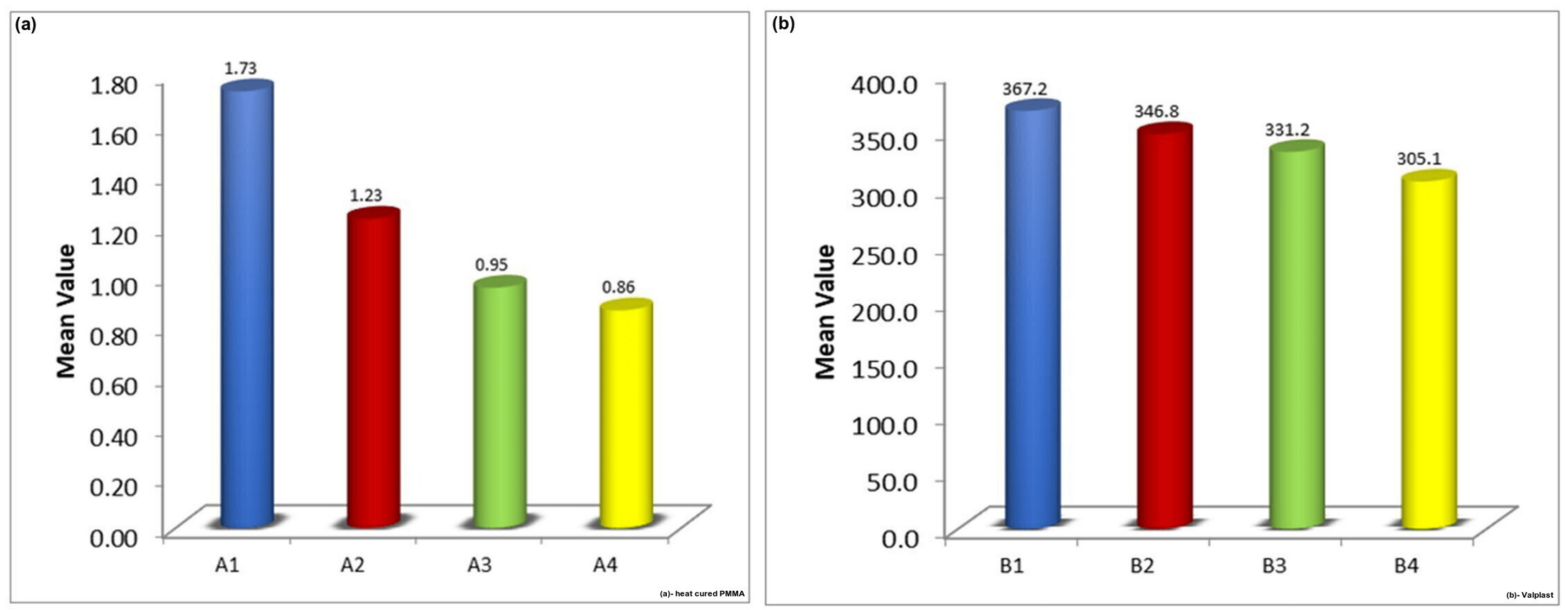
Comparison of impact strength of heat-polymerized polymethylmethacrylate (Acryton H) and polyamide (Valplast) in the four different subgroups. Graphical representation of impact strength values represented as mean value. (a) Heat-cured polymethylmethacrylate. (b) Valplast. A1, B1: Control; A2, B2: Valclean; A3, B3: Polident; A4, B4: Clinsodent.

Graphical representations of the variations are shown as box plots (Figure 11) and bar graphs (Figure 12).

## Discussion

This in vitro study highlights the influence of denture cleansers with varying pH levels on the impact strength of commonly used denture base materials. Clinsodent, an alkaline peroxide-based cleanser, resulted in the most pronounced degradation, particularly in polyamide (Valplast), while Polident, a neutral cleanser, had the least effect. These findings underscore the importance of material-compatible cleanser selection in prosthodontic maintenance protocols to prevent premature mechanical failure of removable prostheses.

The results of this study are in agreement with those of Shah et al. [1], Ragher et al. [2], Nikawa et al. [6], and Hong et al. [7], who also reported that alkaline peroxide-based cleansers caused more degradation in denture base resins. The oxidizing components in Clinsodent (alkaline), particularly sodium perborate, may promote hydrolytic breakdown of the polymer matrix, leading to compromised mechanical integrity. Conversely, Polident, classified as a neutral cleanser, exhibited minimal impact on these properties, which can be due to the presence of acidic agents, such as citric acid, that offset its bleaching action. Similarly, Valclean, being an acidic and peroxide-free cleanser, resulted in only slight alterations. These outcomes are consistent with the findings of Shah et al. [1], Hong et al. [7], Khronghatai [8], and Abubaker et al. [9], who reported reduced denture base changes when using non-peroxide or neutral cleansing agents.

Valplast, a flexible polyamide-based denture material, demonstrated high inherent impact strength; however, it was more susceptible to degradation upon exposure to denture cleansers compared to the conventional heat-cured PMMA (Acryton H). This increased vulnerability is likely due to the higher plasticizer content and residual initiators in Valplast, which are prone to leaching under prolonged aqueous exposure. In the present study, Clinsodent (alkaline peroxide-based) caused the most significant reduction in Valplast’s impact strength, suggesting its unsuitability for flexible resins, whereas Polident (neutral) and Valclean (acidic) caused minimal degradation.

PMMA, though more stable, also showed reduced impact strength due to the migration of polymer chains and leaching of residual monomers, phenomena exacerbated by prolonged water sorption [10,11]. Water acts as a plasticizer, altering the polymer network through hydrogen bonding and expanding the matrix [12,13].

Earlier studies by Pramod et al. [4], Amin et al. [14], and Machado et al. [15] corroborate that repeated cleanser exposure can lead to mechanical degradation through hydrolysis and polymer dissolution. While some cleansers, especially those with extreme pH or alcohol content, can significantly reduce impact strength [3], others, such as Lacalut Dent (oxygenating), have shown minimal adverse effects. Faot et al. [16] emphasized the role of resin composition in fracture resistance, noting that high-impact acrylics perform better under stress. However, some reports suggest that when used according to manufacturer guidelines, chemical cleansers may not significantly alter the mechanical properties of denture base materials [5,17,18]. Thus, the compatibility between cleanser type and denture base material must be considered to preserve long-term prosthesis durability.

Future research should explore the long-term effects of denture cleansers under dynamic intraoral conditions through clinical or in vivo studies. Evaluating additional mechanical properties such as flexural strength, hardness, and surface roughness would provide a more comprehensive understanding of material behavior. Clinically, the study advocates for increased awareness among dental professionals when prescribing denture cleansers, especially for flexible denture materials, to ensure durability and minimize the risk of fracture or deformation during routine use.

While the controlled in vitro setup allowed for standardization and reproducibility, it may not fully replicate the complex intraoral environment, where variables such as saliva composition, thermal cycling, and masticatory forces also influence material behavior. Additionally, only one mechanical property (impact strength) was assessed, and only a single brand per material type was tested. Despite these limitations, the study’s strength lies in its comparative design, standardized sample preparation, and simulation of six-month cleanser use, which adds valuable data to the limited literature on the mechanical effects of chemical denture cleansers.

## Conclusions

Impact strength is an essential property of denture base materials as it determines their ability to withstand sudden functional and accidental loads. A reduction in this property increases the risk of denture fracture, particularly among elderly or medically compromised patients who rely on chemical cleansers for maintaining denture hygiene. The present study demonstrated that exposure to denture cleansers with extreme pH significantly reduced the impact strength of both PMMA and polyamide resins. Among the tested agents, Clinsodent (alkaline) showed the most pronounced degrading effect, especially on polyamide, while Polident (neutral) produced the least change. PMMA displayed greater resistance to cleanser-induced degradation than polyamide, highlighting its superior mechanical stability. These findings emphasize the need for clinicians to recommend denture cleansers based on the type of denture base material used to preserve prosthesis strength, minimize fracture risk, and ensure long-term clinical performance.
